# Experimental and Established Oximes as Pretreatment before Acute Exposure to Azinphos-Methyl

**DOI:** 10.3390/ijms22063072

**Published:** 2021-03-17

**Authors:** Dietrich E. Lorke, Syed M. Nurulain, Mohamed Y. Hasan, Kamil Kuča, Georg A. Petroianu

**Affiliations:** 1 Department of Anatomy and Cellular Biology, College of Medicine and Health Sciences, Khalifa University, Abu Dhabi P.O. Box 127788, United Arab Emirates; 2 Center for Biotechnology, Khalifa University of Science and Technology, Abu Dhabi P.O. Box 127788, United Arab Emirates; 3 Bio Science Department, COMSATS Institute of Information Technology, Bio Sciences Block, CUI, Park Road, Tarlai Kalan, Islamabad 45550, Pakistan; syed.nurulain@comsats.edu.pk; 4 Department of Pharmacology & Therapeutics, College of Medicine and Health Sciences, UAE University, Al Ain P.O. Box 15551, United Arab Emirates; my.baniyas@moe.gov.ae; 5 Department of Chemistry, Faculty of Science, University of Hradec Kralove, Rokitanského 62/26, 500 03 Hradec Kralove, Czech Republic; kamil.kuca@uhk.cz; 6 Department of Pharmacology, College of Medicine and Health Sciences, Khalifa University, Abu Dhabi P.O. Box 127788, United Arab Emirates; Georg.petroianu@ku.ac.ae

**Keywords:** acetylcholine, azinphos-methyl, carbamates, cholinesterase, Cox analysis, obidoxime, organophosphate, pesticide, pralidoxime, prophylaxis, rat

## Abstract

Poisoning with organophosphorus compounds (OPCs) represents an ongoing threat to civilians and rescue personal. We have previously shown that oximes, when administered prophylactically before exposure to the OPC paraoxon, are able to protect from its toxic effects. In the present study, we have assessed to what degree experimental (K-27; K-48; K-53; K-74; K-75) or established oximes (pralidoxime, obidoxime), when given as pretreatment at an equitoxic dosage of 25% of LD_01_, are able to reduce mortality induced by the OPC azinphos-methyl. Their efficacy was compared with that of pyridostigmine, the only FDA-approved substance for such prophylaxis. Efficacy was quantified in rats by Cox analysis, calculating the relative risk of death (RR), with RR=1 for the reference group given only azinphos-methyl, but no prophylaxis. All tested compounds significantly (*p* ≤ 0.05) reduced azinphos-methyl-induced mortality. In addition, the efficacy of all tested experimental and established oximes except K-53 was significantly superior to the FDA-approved compound pyridostigmine. Best protection was observed for the oximes K-48 (RR = 0.20), K-27 (RR = 0.23), and obidoxime (RR = 0.21), which were significantly more efficacious than pralidoxime and pyridostigmine. The second-best group of prophylactic compounds consisted of K-74 (RR = 0.26), K-75 (RR = 0.35) and pralidoxime (RR = 0.37), which were significantly more efficacious than pyridostigmine. Pretreatment with K-53 (RR = 0.37) and pyridostigmine (RR = 0.52) was the least efficacious. Our present data, together with previous results on other OPCs, indicate that the experimental oximes K-27 and K-48 are very promising pretreatment compounds. When penetration into the brain is undesirable, obidoxime is the most efficacious prophylactic agent already approved for clinical use.

## 1. Introduction

Organophosphorus compounds (OPCs), which are synthetic derivatives of phosphoric (organophosphates), phosphonic (organophosphonates), or thiophosphoric/phosphonic acid, are mainly employed in agriculture as pesticides because of their short environmental half-life and superior insecticidal toxicity compared with organochlorines, e.g., DDT [1]. Highly poisonous OPCs, the so-called “nerve agents” tabun, sarin, cyclosarin, soman, venomous agent X (VX), and others have also been developed for combat purposes, and some of them have been misused for terrorist attacks, chemical warfare, and criminal poisonings [2]. In the 1980s, during the Iran-Iraq war, tabun and sarin were used in Basra against Iranian troops and in Hilabjah against the Kurdish population of Northern Iraq [3,4,5,6], causing thousands of fatalities. In the 1990s, the Aum Shinrikyo sect, using sarin and VX, perpetrated three terrorist attacks in the Japanese cities of Tokyo, Osaka, and Matsumoto, resulting in over 20 deaths and a high number of casualties [7,8]. Reports of an improvised bomb produced from pesticides in 1997 [9] and the use of chemical warfare agents during the war in Syria [10] illustrate the ongoing and increasing threat of malicious OPC poisoning, which represents a serious risk not only to affected civilians but also to rescue personal.

The toxicity of OPCs is due to the inhibition of acetylcholinesterase (AChE), the enzyme responsible for breaking down the neurotransmitter acetylcholine (ACh), thereby terminating its action. AChE inhibition leads to accumulation of ACh at cholinergic synapses and increased stimulation of nicotinic and muscarinic receptors both in the peripheral and central nervous systems. As a result, a cholinergic crisis develops with signs and symptoms of excessive bronchial secretion, bronchoconstriction, sweating, lacrimation, vomiting, diarrhea, muscle twitching, paralysis of respiratory muscles, seizures, and coma. Death generally occurs as a result of respiratory failure, seizures, or multiorgan dysfunction [11,12].

The therapeutic outcome of standard post-exposure therapy with atropine blocking muscarinic receptors, benzodiazepines controlling seizures, and oximes reactivating inhibited AChE is unsatisfactory [13,14]. Better results are achieved if reversible cholinesterase inhibitors are administered before exposure (pretreatment, reviewed by [15]). In a series of previous studies with a comparable experimental design, we tested the prophylactic efficacy of a group of reversible cholinesterase inhibitors that are already used clinically for other indications (amiloride, metoclopropramide, methylene blue, physostigmine, pyridostigmine, ranitidine, tacrine, and tiapride) or that have been developed as potential therapeutics (7-methoxitacrine, K-27). These compounds were administered before exposure to a broad range of chemically diverse OPCs, i.e., azinphos-methyl [16], dicrotophos [17], diisopropylfluorophosphate (DFP) [18], ethyl-paraoxon [19,20], methyl-paraoxon [21], and terbufos [22]. Generally, we observed best prophylactic efficacy for physostigmine and K-27, both of which protected significantly better from mortality induced by the majority of OPCs than pyridostigmine [15], the only pretreatment compound approved by the US Food and Drug Administration (FDA) for prophylaxis, when exposure to the nerve agent soman is anticipated [23]. Since physostigmine penetrates the blood brain barrier, its use is not indicated in situations requiring critical decision making due to possible cognitive side effects. K-27 is therefore a very promising alternative, since it hardly enters the brain [24,25,26]. 

The bisquaternary asymmetric pyridinium aldoxime K-27 (Table 1) belongs to a group of experimental oximes that were synthesized and tested in the 2000s [27,28,29,30,31,32,33,34] as an alternative to the established oximes pralidoxime and obidoxime (Table 1), which show disappointing therapeutic results in patients exposed to pesticides and several nerve agents (reviewed by [35]). Given the excellent prophylactic efficacy of K-27, we subsequently tested other experimental K-oximes, including K-48 (Table 1), a different bisquaternary asymmetric pyridinium aldoxime with only one functional aldoxime group in position 4 of the pyridine ring [36,37], as well as K-53, K-74, and K-75 (Table 1), bispyridinium oximes with two aldoxime groups [38,39]. 

Like other oximes, in addition to their enzyme-reactivating ability, K-oximes also display some AChE inhibitory activity [40]. When administered before the OPC paraoxon, the best protection from paraoxon-induced toxicity was observed for K-48 pretreatment, which was significantly more efficacious than the FDA approved pretreatment with pyridostigmine [41]. Since oxime efficacy depends on the OPC, oximes need to be tested in exposure to different chemically diverse OPCs. The present study has therefore been undertaken in order to test if and to what degree the experimental (K-27, K-48, K-53, K-74, K-75) and established oximes (pralidoxime, obidoxime) can protect from the toxic effects of azinphos-methyl (Table 1), an organophosphorothionate (thion) that is employed worldwide as a pesticide.

## 2. Results

### 2.1. Mortality Rates

The survival of the experimental animals depended on the azinphos-methyl dosage, the time point, and the substance employed for prophylaxis. The mortality of rats that had only received 5 µmol azinphos-methyl and no pretreatment increased from 71% after 10 min to 75% after 24 h (group 1), whereas the mortality of animals injected with 15 µmol azinphos-methyl was 92% after 10 min and 100% from 1 h onwards (Table 2). Some of the animals showed signs typical of cholinergic excitation. Pretreatment with pyridostigmine or any of the oximes examined decreased these mortality rates, e.g., to 46% when K-27 was given 30 min before exposure to 15 µmol azinphos-methyl (group 5). None of the control rats that only received equitoxic doses of the prophylactic agent but no azinphos-methyl died, corresponding to a mortality rate of 0%. No signs of cholinergic excitation were observed.

### 2.2. Cox Survival Analysis

**Figure 1:**
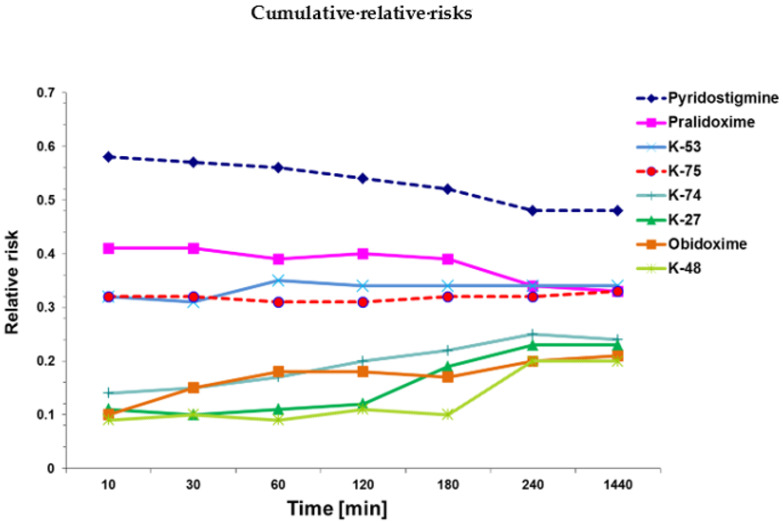
Relative risk (RR) of death, estimated by Cox [42] analysis in animals exposed to intraperitoneal (i.p.) injections of azinphos-methyl and adjusted for azinphos-methyl dose (high/low) for each of the time points examined (10 min, 30 min, 1 h, 2 h, 3 h, 4 h, 24 h, 48 h). The acetylcholinesterase (AChE) inhibitor pyridostigmine or the oxime-type AChE reactivators pralidoxime, obidoxime, K-27, K-48, K-53, K-74 or K-75 were administered prophylactically 30 min before azinphos-methyl exposure, and the protection conferred by these compounds was compared with the reference group (azinphos-methyl alone, no pretreatment: RR = 1). The injected dose for pretreatment was approximately ¼ the LD_01_. The most successful prophylactic compound was K-48, reducing the relative risk of death to about 8% (RR = 0.08) after 10 min and to about 20% (RR = 0.20) after 48 h (1440 min). Pretreatment with pyridostigmine was the least efficacious, reducing azinphos-methyl-induced mortality only to about 58% after 10 min and to 50% after 48 h.

The relative risk of death (RR) observed 10 min, 30 min, 1 h, 2 h, 3 h, 4 h, 24 h, and 48 h after azinphos-methyl exposure estimated by Cox analysis [42] in pretreated animals is depicted in Figure 1. The RR compared with the reference group that had only received azinphos-methyl but no prophylaxis (RR = 1) and was adjusted for azinphos-methyl dose (high/low). The statistical analysis (Table 4) of the different pretreatment protocols is based on the cumulative relative risk, i.e., the area under the RR time curve (Figure 1).

Statistical comparison revealed that pretreatment with all tested compounds significantly (*p* ≤ 0.05) reduced azinphos-methyl-induced mortality (reference group: RR = 1). The most efficacious prophylactic agents were K-27 and K-48 (Table 3), reducing the relative risk of death to 20% (K-48) or 23% (K-27), respectively, which was significantly (*p* ≤ 0.05) superior to pretreatment with most of the other tested compounds, i.e., pyridostigmine (RR = 0.52), pralidoxime (RR = 0.37), and K-75 (RR = 0.37). Another very efficacious prophylactic compound was obidoxime (RR=0.21), which was significantly (*p* ≤ 0.05) more efficacious than pyridostigmine and pralidoxime (Table 4). 

The second best group of prophylactic compounds consisted of pralidoxime (RR = 0.37), K-74 (RR = 0.26), and K-75 (RR = 0.35), which were significantly (*p* ≤ 0.05) more efficacious than pyridostigmine. Pretreatment with K-53 (RR = 0.37) was in the same order of magnitude, although not significantly different from pyridostigmine prophylaxis. The least efficacious pretreatment agent was pyridostigmine (RR = 0.52), still significantly (*p* ≤ 0.05) reducing azinphos-methyl-induced mortality.

## 3. Discussion

We have previously been able to demonstrate that the experimental oxime K-27, when administered 30 min before exposure to a variety of chemically diverse OPCs, generally has significantly better efficacy than pyridostigmine [15,19,26], the only compound approved by the FDA for pretreatment when threat of soman exposure exists [23]. Moreover, we could show that a group of experimental (K-48, K-53, K-74, K-75) and established oximes (pralidoxime, obidoxime), when given as pretreatment, significantly reduced paraoxon-induced mortality, with K-48 affording significantly better protection than pyridostigmine [41]. The present study has therefore been undertaken in order to assess if these oximes also protect from mortality induced by another chemically different OPC, azinphos-methyl. These oximes have previously been shown to efficiently reduce azinphos-methyl-induced mortality when given as post-exposure antidote therapy [43].

The OPC azinphos-methyl [O,O-Dimethyl-S-(4-oxo-3H-1,2,3- benzotriazin-3-yl)methyl-dithiophosphat], the active component in numerous commercially available pesticide formulations, e.g., Bay 9027, Bay 17147, Carfene, Cotnion-methyl, Cotnion, Crysthyron, Gusathion, Gusathion-M, Guthion, Metriltrizotion, and R-1852 [44], is an organophosphorothionate (thion) that is globally employed as a broad-range insecticide [45,46,47]. It hardly inhibits AChE in its phosphorothionate (thion) form, but in vivo it is quickly desulfurized to the very poisonous oxon (phosphate triester) [48]. CYP1A2 is the main cytochrome involved in this hepatic bioconversion, but when exposure is extensive, other cytochromes, particularly CYP3A4, also participate. This bioactivation happens rapidly: in vivo symptoms characteristic of a cholinergic crisis are seen in animal experiments about 5 min after oral administration, and the conversion of the thion to the oxon form takes less than 10 min in an in vitro liver slice model [49,50]. Fast bioactivation is also indicated by our own earlier [16,43] and present reference data, demonstrating death within 10 min of 92% of the experimental animals exposed to 10–15 µmol azinphos-methyl, when no additional treatment is given.

When considering a compound for pretreatment, in many cases the exact nature of the OPC is unknown. The best pretreatment agent is therefore efficacious against a broad range of chemically diverse OPCs. We have previously tested the efficacy of novel and established oximes when administered before ethyl-paraoxon, i.e., di-ethyl-4-nitrophenyl phosphate [41]. Azinphos, a di-methyl di-thiophosphate with a benzotriazin residue, is an OPC with a structure that is clearly distinguished from that of paraoxon. In the present study, azinphos was not chosen because of its pesticidal activity but in order to test if any of the tested compounds has a broad-spectrum prophylactic efficacy against chemically diverse OPCs.

When evaluating the efficacy of different pretreatment compounds, they have to be administered in biologically defined dosages. We have previously explained in detail [15,18,20] why we are of the opinion that dosing according to in vivo parameters (25% of LD_01_, i.e., the dose killing 1% of the animals) best reflects the clinical reality, since equitoxicity based on AChE inhibition in vitro (IC_50_) would ignore toxicities not related to AChE inhibition, thereby producing false negative results. Overall, signs of acute toxicity of established and experimental oximes are only observed at very high dosages. Available data also indicate that long term toxic effects are highly unlikely [26].

The present study demonstrates that all tested oximes and the carbamate pyridostigmine, when given as pretreatment, significantly reduce azinphos-methyl-induced mortality. Moreover, the efficacy of all tested experimental and established oximes except K-53 was significantly superior to the FDA-approved compound pyridostigmine. Best protection was observed for the two experimental oximes K-27 and K-48, which reduced the RR to 0.20 (K-48) and 0.23 (K-27), which was significantly superior to pralidoxime, K-75, and pyridostigmine. These results corroborate the results of an earlier study demonstrating that K-27, when given as pretreatment, is significantly more efficacious than pyridostigmine in protecting from azinphos-methyl-induced mortality [16] and extends these findings to other experimental oximes not yet tested, i.e., K-48, K-53, K-74, K-75. Lucic Vrdoljak also tested the prophylactic efficacy of oximes K-48 and K-33, but their study was designed very differently. Oximes were administered at a much higher dosage (25% of LD_50_ compared to 25% of LD_01_ in our study) 15 min before subcutaneous injections of the nerve agent tabun, followed by treatment with atropine plus oxime (K-27, K-48, K-33, trimedoxime, or HI-6) 1 min after tabun exposure [51]. In their experiment, better protection was observed for K-48 compared to K-33, when used as pretreatment.

Regarding the mechanism of action, several theories have been discussed previously [15,17,22]. In the first publications describing that pretreatment with the reversible AChE inhibitors physostigmine and other carbamates protects against the toxic effects of the OPC DFP both in vitro [52] and in vivo [53], the authors speculated that the reversible AChE inhibitor temporarily occupies the catalytic site of the enzyme, thereby sheltering it from being irreversibly inhibited by the OPC. The validity of this mechanism has just recently been demonstrated again in vitro and in vivo for a novel slow-binding AChE inhibitor [54]. It may also apply to oximes, since, in addition to reactivating AChE, they also inhibit this enzyme, although to a much lesser degree than OPCs [40]. 

Another theory was brought forward by Soreq and Seidmann, who demonstrated an increase in AChE synthesis at the mRNA and protein level in mouse brain slices incubated with the AChE inhibitors DFP and pyridostigmine [55,56,57]. A third mechanism may come into play for oximes: pretreatment with oximes may also protect from OPC toxicity by directly reactivating the inhibited AChE molecule. K-27 and K-48 reach their maximum plasma concentrations 5 (K-27) to 15 min (K-48) after intramuscular injection into rats and have a plasma half-life of about 60 min [24]. It is therefore conceivable that 30 min after i.p. injection of K-27 and K-48, there is still sufficient oxime in the plasma to reactivate OPC-inhibited AChE. It seems, however, unlikely that this is the only mechanism, since we previously demonstrated that K-53 is more efficacious than K-27 and K-48 when given as post-treatment after azinphos-methyl exposure [43]. When given as pretreatment in our present study, however, K-53 was much less efficacious.

K-27 and K-48, which are bisquaternary asymmetric pyridinium aldoximes with one functional aldoxime group, have very promising reactivation characteristics of OPC-inhibited AChE [26]. In vitro (human cell lines) and in vivo testing in rodents indicate low toxicity of both oximes [26,58]. K-27 and K-48 hardly pass the blood brain barrier, only 2% of K-27 and 5% of K-48 injected intramuscularly enter the brain [24,25]. Passage into the brain is not desired for compounds given as pretreatment, because they may impair performance in situations that require critical decision making. This gives both K-27 and K-48 a definite edge over physostigmine as prophylactic compounds. K-27 and K-48 have not yet been approved for clinical use. Our data also demonstrate that the efficacy to protect from both azinphos and paraoxon [41] exposure of the established oxime obidoxime (RR = 0.21) is in the same order of magnitude as that of K-27 and K-48, which is significantly superior to pyridostigmine. The passage of obidoxime through the blood-brain barrier is also relatively restricted, only about 5.5 % of plasma concentration reaches the brain [24]. Since K-27 and K-48 have not yet obtained approval, obidoxime is therefore the most promising established pretreatment compound already authorized for clinical use.

## 4. Materials and Methods

### 4.1. Chemicals

Azinphos-methyl stock solution (100 mmol/L) was prepared in dry acetone. The working solution for intraperitoneal (i.p.) application was prepared ex tempore by diluting stock solution with saline shortly before application. Azinphos-methyl (Azinphos-methyl PESTANAL^®^, analytical standard), pyridostigmine (Pyridostigmine bromide, product number: P9797, purity [HPLC] ≥98%), and pralidoxime chloride (Pyridine-2-aldoxime methochloride, product number: P9053, purity ≥99%) were purchased from Sigma-Aldrich Chemie (Sigma-Aldrich Chemie GmbH, Steinheim, Germany), while obidoxime (Obidoxime chloride, product number: 51063, purity [HPLC] ≥95%) was purchased from Fluka Chemical AG (Buchs, Switzerland). The other oximes (K-27, K-48, K-53, K-74, and K-75) were synthesized in the Department of Toxicology at the Faculty of Military Health Sciences (University of Defence, Hradec Kralove, Czech Republic) according to Kuca et al. [37] and tested for purity by thin-layer chromatography (TLC) and high-performance liquid chromatography (HPLC) as described in detail by Jun et al. [59,60]. The water was distilled and de-ionized.

### 4.2. Experimental Animals

During the entire experiment, the “Guiding Principles in the Care of and Use of Laboratory Animals” (Council of The American Physiological Society) were observed. All studies were performed with the approval of the relevant institutional review board (Faculty of Medicine and Health Sciences Animal Research Ethics Committee; AE/18/09).

The original stock of Wistar rats was purchased from Harlan Laboratories (Harlan Laboratories, Oxon, England). The animals used in the present studies were bred from the original stock at the Animal Facilities of the College of Medicine and Health Sciences, UAE University. Adult male rats (average weight ± SD: 259 ± 13 g; 95% confidence interval: 258–260 g) were kept in polypropylene cages (43 × 22.5 × 20.5 cm^3^; six rats/cage) in climate- and access-controlled rooms (23 ± 1 °C; 50 ± 4% humidity). The day/night cycle was 12 h/12 h. Food and water were available ad libitum. The food was standard maintenance diet for rats purchased from Emirates Feed Factory (Abu Dhabi, UAE).

#### 4.2.1. Choice of Dosage for Pretreatment

Regarding pretreatment dosages, 25% of LD_01_ [18] was considered a quantity well tolerated by the experimental animals, and therefore the following dosages were administered for pretreatment (Table 4):

- Reference group: only azinphos-methyl exposure.
- Pyridostigmine: 1 µmol/rat = 0.26 mg/rat (= 1.0 mg/kg average body weight).
- Pralidoxime: 30 µmol/rat = 5.2 mg/rat (= 20 mg/kg average body weight).
- Obidoxime: 25 µmol/rat = 9.0 mg/rat (=35 mg/kg average body weight).
- K-27: 60 µmol/rat = 26.8 mg/rat (=103 mg/kg average body weight).
- K-48: 25 µmol/rat = 11.5 mg/rat (=44 mg/kg average body weight).
- K-53: 3 µmol/rat = 1.37 mg/rat (=5.3 mg/kg average body weight).
- K-74: 3 µmol/rat = 1.38 mg/rat (=5.3 mg/kg average body weight).
- K-75: 3 µmol/rat = 1.37 mg/rat (=5.3 mg/kg average body weight).

#### 4.2.2. Pretreatment and Azinphos-Methyl Exposure

When performing a Cox analysis [42], the therapeutic efficacies of different therapeutic agents are tested and compared against a toxic compound administered at different dosages. Based on LD values previously determined [43], we chose to administer a dosage range between the LD_75_ and the lowest dosage killing all animals (LD_100_). Consequently, in the experimental groups, animals received i.p. injections of azinphos-methyl (MW 317.3), in a dosage of either 5 µmol = 1.59 mg (6.14 mg/kg average body weight ≈ LD_75_), 10 µmol = 3.18 mg (12.28 mg/kg average body weight ≈ twice LD_75_), or 15 µmol = 4.77 mg (18.42 mg/kg average body weight ≈ three times LD_75_), diluted in 500 µl saline solution (Table 4). For each dosage, there were 9 groups of rats; the experiments were repeated four times (4 cycles; 6 rats/cycle). The reference group (azinphos-methyl) was given azinphos-methyl i.p. alone. Groups 2–9 received i.p. injections of the prophylactic agents (pyridostigmine, pralidoxime, obidoxime, K-27, K-48, K-53, K-74, and K-75, diluted in 500 µl saline solution) and an azinphos-methyl injection 30 min later. The pretreatment compound and azinphos-methyl were injected at two anatomically distinct sites, thereby minimizing the risk of interaction between the pretreatment agent and the OPC in the peritoneal cavity.

**Table 4:** Molecular weights, dosages, cholinesterase (AChE) inhibition, and LD values of the compounds administered. Column 2 lists their molecular weights, columns 3–5 the injected doses. Values are given in µmol/animal (column 3), in mg/animal (column 4), and in mg/kg average body weight (column 5). Column 6 lists their concentration necessary to inhibit 50% of human red blood cell AChE activity (IC_50_), column 7 the IC_50_ for AChE inhibition determined in rat blood [61], and column 8 their LD_50_ and LD_01_ values for intraperitoneal (i.p.) application in rats [18]. The azinphos-methyl dose injected i.p. ranges from 5 µmol ≈ LD_75_ (first value) to 15 µmol ≈ LD_100_ (third value); the dosage of the compounds administered prophylactically (pyridostigmine, pralidoxime, obidoxime, K-27, K-48, K-53, K-74, K-75) before azinphos-methyl exposure was approximately ¼ of the LD_01_. * Compound must be metabolized to bioactive oxon form. NA: not assessed.

	Molecular Weight	Injected Dose (µmol/rat)	Injected Dose (mg/rat)	Injected Dose (mg/kg Average Body Weight)	IC_50_ Human (µM]	IC_50_ Rat (µM)	LD_50_/LD_01_ (µmol/rat)
Azinphos-methyl	317.3	5, 10, 15	1.59, 3.18, 4.77	6.14, 12.28, 18.42	189 *	NA	3.2/0.4 *
Pyridostigmine	172.60	30	0.26	1.0	0.33	NA	7.2/3.7
Pralidoxime	172.60	30	5.2	20	592	412	180/117
Obidoxime	359.21	25	9.0	35	702	193	132/107
K-27	446.16	60	26.8	103	414	1054	350/250
K-45	460.16	25	11.5	44	461	643	140/110
K-53	458.15	3	1.37	5.3	115	83	21/13
K-74	460.16	3	1.38	5.3	103	66	28/13
K-75	458.15	3	1.37	5.3	63	101	51/13

The animals were monitored for 48 h, and mortality was recorded at 10 min, 30 min, and 1, 2, 3, 4, 24, and 48 h. There were 8 control groups, consisting of 6 rats each, which received only the prophylactic agent but no azinphos-methyl injections. 

### 4.3. Statistical Analysis

Statistical analysis was performed on the mortality data of 4 cycles. Mortality data were compared, and for each of the eight time points, the respective hazards ratios (relative risks of death) were estimated using the Cox proportional hazards model [42]. Both azinphos-methyl dose (10 and 15 µmol/rat, respectively, with 5 µmol as the reference category) and group, i.e., type of pretreatment (with group 1, i.e., azinphos-methyl only without pretreatment, as the reference category) were treated as categorical variables.

Subsequently, the area under the RR-time curve was determined and pair-wise comparisons (Mann-Whitney U-Test) were performed in order to determine the most protective reactivator. No Bonferroni correction for multiple comparisons was applied, and *p* ≤ 0.05 was considered significant. The IBM SPSS^®^ Statistics 25.0 (IBM Corp. Armonk, NY, USA) software package was used for all statistical evaluation.

## 5. Conclusions

All tested experimental (K-27, K-48, K-53, K-74, K-75) and established (pralidoxime, obidoxime) oximes protected significantly better from azinphos-methyl toxicity than the FDA-approved compound pyridostigmine. The experimental oximes K-27 and K-48 afforded the best protection and are a promising alternative to pyridostigmine when entry into the brain is unwarranted. Since K-27 and K-48 have not yet been approved for clinical use, obidoxime is a very efficacious prophylactic alternative already used clinically. 

## Figures and Tables

**Table 1:** Chemical structures of the organophosphate pesticide azinphos-methyl and of the compounds tested as prophylaxis before azinphos-methyl exposure. Pyridostigmine is a strong inhibitor of acetylcholinesterase (AChE) that does not penetrate the blood-brain barrier. Up to now, it is the only substance approved by the American Food and Drug Agency (FDA) for pretreatment when soman-exposure is imminent. Pralidoxime and obidoxime are oxime-type AChE reactivators that are already used in the clinical therapy of organophosphorus compound (OPC) poisoning. K-27, K-48, K-53, K-74, and K-75 are experimental oximes synthesized and tested during the last 15 years by the research group of Kamil Kuca et al. in order to improve the fatal outcome of OPC exposure.

Substance	Structure
**Azinphos-methyl**	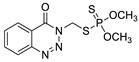
**Pyridostigmine**	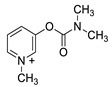
**Pralidoxime**	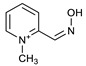
**Obidoxime**	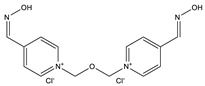
**K-27**	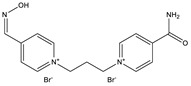
**K-48**	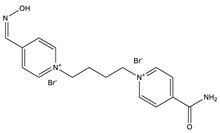
**K-53**	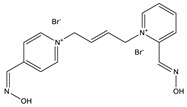
**K-74**	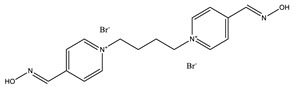
**K-75**	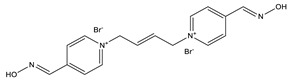

**Table 2:** Mortality of experimental animals exposed to intraperitoneal (i.p.) injections of azinphos-methyl in a dosage of 5 (first value), 10 (second value), or 15 µmol (third value). Listed are the proportions of dead animals in percent at each time point (10 min, 30 min, 1 h, 2 h, 3 h, 4 h, 24 h, and 48 h after azinphos-methyl injection) for untreated rats (group 1, reference: azinphos-methyl only) and for animals pretreated with pyridostigmine, established oximes (pralidoxime, obidoxime), or experimental K-oximes (K-27, K-48, K-53, K-74, K-75) before azinphos-methyl exposure. The dose injected for pretreatment was approximately ¼ of the LD_01_.

Groups (G)	10 min	30 min	1 h	2 h	3 h	4 h	24 h	48 h
G1: Azinphos-methyl only	71/92/92	71/92/96	71/92/100	71/92/100	71/92/100	71/92/100	75/92/100	75/92/100
G2: Pyridostigmine pretreatment	29/50/71	42/54/75	42/54/79	42/58/83	42/58/88	42/58/88	46/58/88	46/58/88
G3: Pralidoxime pretreatment	21/33/54	21/50/58	21/50/67	25/50/71	25/63/71	25/63/71	25/63/71	25/63/71
G4: Obidoxime pretreatment	4/8/21	4/8/21	4/8/42	4/29/42	4/29/54	4/29/54	17/46/63	17/46/67
G5: K-27 pretreatment	21/0/4	21/4/13	21/4/13	21/4/25	29/4/29	29/42/29	50/46/46	50/50/46
G6: K-48 pretreatment	0/8/8	8/13/13	8/13/21	8/13/21	8/13/38	8/13/38	25/50/58	25/54/58
G7: K-53 pretreatment	13/33/29	29/33/42	29/33/50	29/46/63	29/50/63	29/50/63	38/54/71	38/54/71
G8: K-74 pretreatment	0/8/17	17/8/25	17/8/38	17/13/46	17/33/50	17/50/50	33/58/54	33/58/54
G9: K-75 pretreatment	4/17/33	33/21/50	38/21/58	38/25/58	38/38/58	42/42/58	50/50/63	50/58/67

**Table 3:** Survival analysis [42] of the cumulative relative risk (RR) of death, including 95% confidence interval (CI), of animals injected with azinphos-methyl (5, 10 or 15 µmol) intraperitoneally (i.p.). Values are adjusted for azinphos-methyl dose (high/low). The cumulative RR was assessed by determining the area under the RR-time curve (see Figure 1) for pre-exposure treatment with pyridostigmine, pralidoxime, obidoxime, K-27, K-48, K-53, K-74, or K-75. The injected dose was approximately ¼ of the LD_01_. Group 1, i.e., only azinphos-methyl and no pretreatment, was the reference category (RR = 1). Listed are mean values ± standard deviations (SD). Statistical differences relative to the reference group (only azinphos-methyl and no pretreatment) were tested by the Mann-Whitney U-Test, and a *p* value ≤ 0.05 was considered significant. ^a^: mortality significantly decreased compared with pyridostigmine; ^b^: mortality significantly decreased compared with pralidoxime; ^c^: mortality significantly decreased compared with K-75.

Groups	Relative Risk (RR)	95% CI	*p*-Value
Azinphos-methyl only	1	reference	reference
Pyridostigmine + azinphos	0.52 ± 0.10	0.36–0.68	≤0. 01
Pralidoxime + azinphos	0.37 ± 0.03	0.33–0.41	≤0. 01 ^a^
Obidoxime+ azinphos	0.21 ± 0.09	0.06–0.36	≤0. 01 ^a, b^
K-27 + azinphos	0.23 ± 0.02	0.20–0.25	≤0.01 ^a, b, c^
K-48 + azinphos	0.20 ± 0.03	15–0.24	≤0.01 ^a, b, c^
K-53 + azinphos	0.37 ± 0.14	0.14–0.59	≤0.01
K-74 + azinphos	0.26 ± 0.10	0.09–0.42	≤0.01 ^a^
K-75 + azinphos	0.35 ± 0.03	0.30–0.39	≤0.01 ^a^

a. *p* ≤ 0.05 compared with pyridostigmine; b. *p* ≤ 0.05 compared with pralidoxime; c. *p* ≤ 0.05 compared with K-75.
